# Isoamyl 2-(5-bromo-3-methyl­sulfin­yl-1-benzofuran-2-­yl)acetate

**DOI:** 10.1107/S1600536809020613

**Published:** 2009-06-06

**Authors:** Hong Dae Choi, Pil Ja Seo, Byeng Wha Son, Uk Lee

**Affiliations:** a Department of Chemistry, Dongeui University, San 24 Kaya–dong Busanjin–gu, Busan 614–714, Republic of Korea; b Department of Chemistry, Pukyong National University, 599–1 Daeyeon 3–dong, Nam–gu, Busan 608–737, Republic of Korea

## Abstract

In the title compound, C_16_H_19_BrO_4_S, the O atom and the methyl group of the methyl­sulfinyl substituent lie on opposite sides of the plane of the benzofuran fragment. The crystal structure exhibits aromatic π–π inter­actions between the benzene rings of adjacent mol­ecules [centroid–centroid distance = 3.643 (2) Å] and nonclassical C—H⋯O hydrogen bonds.

## Related literature

For the crystal structures of similar alkyl 2-(5-bromo-3-methyl­sulfin­yl-1-benzofuran-2-yl)acetate derivatives. see: Choi *et al.* (2009*a* ▶,*b* ▶). For the biological and pharmacological activity of benzofuran compounds, see: Howlett *et al.* (1999 ▶); Ward (1997 ▶).
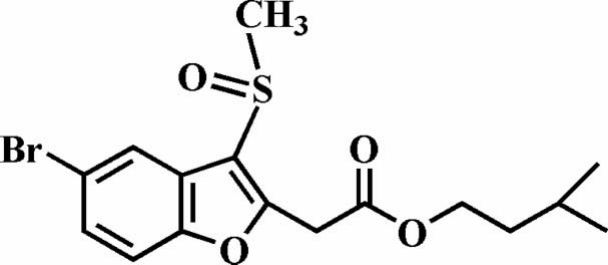


## Experimental

### 

#### Crystal data

C_16_H_19_BrO_4_S
*M* *_r_* = 387.28
Triclinic, 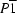

*a* = 8.3704 (4) Å
*b* = 10.2956 (6) Å
*c* = 10.524 (1) Å
α = 99.977 (1)°
β = 105.230 (1)°
γ = 100.681 (1)°
*V* = 836.04 (10) Å^3^
*Z* = 2
Mo *K*α radiation
μ = 2.60 mm^−1^
*T* = 173 K
0.60 × 0.40 × 0.10 mm

#### Data collection

Bruker SMART CCD diffractometer
Absorption correction: multi-scan *SADABS* (Sheldrick, 1999 ▶) *T*_min_ = 0.302, *T*_max_ = 0.769
7179 measured reflections
3579 independent reflections
3304 reflections with *I* > 2σ(*I*)
*R*_int_ = 0.024

#### Refinement

*R*[*F* ^2^ > 2σ(*F* ^2^)] = 0.027
*wR*(*F* ^2^) = 0.072
*S* = 1.04
3579 reflections
202 parameters
H-atom parameters constrained
Δρ_max_ = 0.35 e Å^−3^
Δρ_min_ = −0.59 e Å^−3^

### 

Data collection: *SMART* (Bruker, 2001 ▶); cell refinement: *SAINT* (Bruker, 2001 ▶); data reduction: *SAINT*; program(s) used to solve structure: *SHELXS97* (Sheldrick, 2008 ▶); program(s) used to refine structure: *SHELXL97* (Sheldrick, 2008 ▶); molecular graphics: *ORTEP-3* (Farrugia, 1997 ▶) and *DIAMOND* (Brandenburg, 1998 ▶); software used to prepare material for publication: *SHELXL97*.

## Supplementary Material

Crystal structure: contains datablocks I. DOI: 10.1107/S1600536809020613/cv2569sup1.cif

Structure factors: contains datablocks I. DOI: 10.1107/S1600536809020613/cv2569Isup2.hkl

Additional supplementary materials:  crystallographic information; 3D view; checkCIF report

## Figures and Tables

**Table 1:** Hydrogen-bond geometry (Å, °)

*D*—H⋯*A*	*D*—H	H⋯*A*	*D*⋯*A*	*D*—H⋯*A*
C3—H3⋯O4^i^	0.93	2.56	3.437 (2)	157
C5—H5⋯O3^ii^	0.93	2.51	3.383 (2)	157
C9—H9*A*⋯O4^iii^	0.97	2.26	3.192 (2)	162
C9—H9*B*⋯O1^iv^	0.97	2.61	3.541 (2)	160

Symmetry codes: (i) 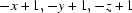; (ii) 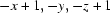; (iii) 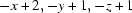; (iv) 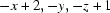.

**Fig. 1.**
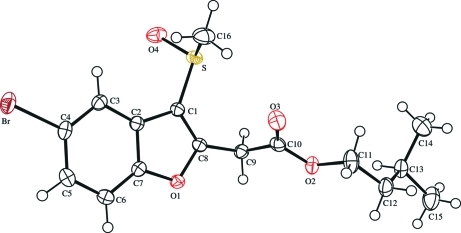
The molecular structure of the title compound with the atom numbering scheme. Displacement ellipsoids are drawn at the 50% probability level.

**Fig. 2.**
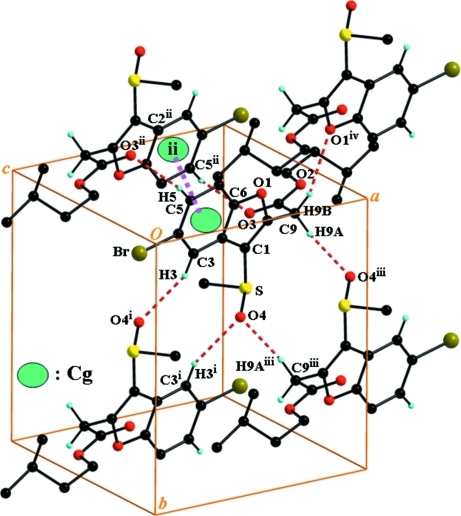
The π···π and C–H···O interactions (dotted lines) in the title compound. Cg denotes the ring centroids. [Symmetry code: (i) -x + 1, -y + 1, -z + 1; (ii) -x + 1, -y, -z + 1; (iii) -x + 2, -y + 1, -z + 1; (iv) -x + 2, -y, -z + 1.]

## supplementary crystallographic information

### Comment

The benzofuran ring system has attracted considerable interest in view of their biological and pharmacological properties (Howlett *et al.*, 1999; Ward, 1997). This work is related to our communications on the synthesis and structures of alkyl 2-(5-bromo-3-methylsulfinyl-1-benzofuran-2-yl)acetate analogues, *viz*. butyl 2-(5-bromo-3-methylsulfinyl-1-benzofuran-2-yl)acetate (Choi *et al.*, 2009*a*) and propyl 2-(5-bromo-3-methylsulfinyl-1-benzofuran-2-yl)acetate (Choi *et al.*, 2009*b*). Here we report the crystal structure of the title compound, isoamyl 2-(5-bromo-3-methyllsulfinyl-1-benzofuran-2-yl)acetate (Fig. 1).

The benzofuran unit is essentially planar, with a mean deviation of 0.015 (1) Å from the least-squares plane defined by the nine constituent atoms. The crystal packing (Fig. 2) is stabilized by aromatic π···π interactions between the benzene rings from the adjacent molecules. The Cg···Cg^ii^ distance of 3.643 (2) Å (Cg is the centroid of C2-C7 benzene ring. symmetry code as in Fig. 2). The molecular packing is further stabilized by four different types of non-calssical C–H···O hydrogen bonds; the first between a benzene H atom and the S=O unit, the second between a benzene H atom and the C═O unit, the third between an H atom of the methylene group bonded to the carboxylate C atom and the S═O unit, the fourth between an H atom of the methylene group bonded to the carboxylate C atom and the furan O atom, respectively (Table 1 and Fig. 2; symmetry code as in Fig. 2).

### Experimental

The 77% 3-chloroperoxybenzoic acid (247 mg, 1.1 mmol) was added in small portions to a stirred solution of isoamyl 2-(5-bromo-3-methylsulfanyl-1-benzofuran-2-yl)acetate (371 mg, 1.0 mmol) in dichloromethane (30 ml) at 273 K. After being stirred for 4 h at room temperature, the mixture was washed with saturated sodium bicarbonate solution and the organic layer was separated, dried over magnesium sulfate, filtered and concentrated in vacuum. The residue was purified by column chromatography (hexane-ethyl acetate,1:2 v/v) to afford the title compound as a colorless solid [yield 79%, m.p. 387-388 K; Rf = 0.61 (hexane-ethyl acetate, 1;2 v/v )]. Single crystals suitable for X-ray diffraction were prepared by evaporation of a solution of the title compound in ethyl acetate at room temperature. Spectroscopic analysis: ^1^H NMR (CDCl_3_, 400 MHz) δ 0.91 (d, J = 6.6 Hz, 6H), 1.51-1.57 (m, 1H), 1.62-1.69 (m, 2H), 3.07 (s, 3H), 4.03 (s, 2H), 4.18 (t, J = 6.96 Hz, 2H), 7.39 (d, J = 8.8 Hz, 1H), 7.49 (dd, J = 8.8 Hz and J = 2.2 Hz, 1H), 8.09 (d, J = 1.84 Hz, 1H); EI-MS 388 [M+2], 386 [M^+^].

### Refinement

All H atoms were geometrically positioned and refined using a riding model, with C–H = 0.93 Å for the aryl, 0.97 Å for the methylene, 0.98 Å for the methine, and 0.96 Å for the methyl H atoms. *U*iso(H) = 1.2*U*eq(C) for the aryl, methine and methylene H atoms, and 1.5*U*eq(C) for methyl H atoms.

### Crystal data

**Table:**

C_16_H_19_BrO_4_S	*Z* = 2
*M**_r_* = 387.28	*F*(000) = 396
Triclinic, *P*1	*D*_x_ = 1.538 Mg m^−^^3^
Hall symbol: -p 1	Mo *K*α radiation, λ = 0.71073 Å
*a* = 8.3704 (4) Å	Cell parameters from 5367 reflections
*b* = 10.2956 (6) Å	θ = 2.1–28.2°
*c* = 10.524 (1) Å	µ = 2.60 mm^−^^1^
α = 99.977 (1)°	*T* = 173 K
β = 105.230 (1)°	Block, colourless
γ = 100.681 (1)°	0.60 × 0.40 × 0.10 mm
*V* = 836.04 (10) Å^3^	

### Data collection

**Table:**

Bruker SMART CCD diffractometer	3579 independent reflections
Radiation source: fine-focus sealed tube	3304 reflections with *I* > 2σ(*I*)
graphite	*R*_int_ = 0.024
Detector resolution: 10.0 pixels mm^-1^	θ_max_ = 27.0°, θ_min_ = 2.6°
φ– and ω–scans	*h* = −10→10
Absorption correction: multi-scan *SADABS* (Sheldrick, 1999)	*k* = −13→13
*T*_min_ = 0.302, *T*_max_ = 0.769	*l* = −13→13
7179 measured reflections	

### Refinement

**Table:**

Refinement on *F*^2^	Primary atom site location: structure-invariant direct methods
Least-squares matrix: full	Secondary atom site location: difference Fourier map
*R*[*F*^2^ > 2σ(*F*^2^)] = 0.027	Hydrogen site location: difference Fourier map
*wR*(*F*^2^) = 0.072	H-atom parameters constrained
*S* = 1.04	*w* = 1/[σ^2^(*F*_o_^2^) + (0.0362*P*)^2^ + 0.2834*P*] where *P* = (*F*_o_^2^ + 2*F*_c_^2^)/3
3579 reflections	(Δ/σ)_max_ < 0.001
202 parameters	Δρ_max_ = 0.35 e Å^−^^3^
0 restraints	Δρ_min_ = −0.59 e Å^−^^3^

### Special details

**Table:**

Geometry. All esds (except the esd in the dihedral angle between two l.s. planes) are estimated using the full covariance matrix. The cell esds are taken into account individually in the estimation of esds in distances, angles and torsion angles; correlations between esds in cell parameters are only used when they are defined by crystal symmetry. An approximate (isotropic) treatment of cell esds is used for estimating esds involving l.s. planes.
Refinement. Refinement of F^2^ against ALL reflections. The weighted R-factor wR and goodness of fit S are based on F^2^, conventional R-factors R are based on F, with F set to zero for negative F^2^. The threshold expression of F^2^ > 2sigma(F^2^) is used only for calculating R-factors(gt) etc. and is not relevant to the choice of reflections for refinement. R-factors based on F^2^ are statistically about twice as large as those based on F, and R- factors based on ALL data will be even larger.

### Fractional atomic coordinates and isotropic or equivalent isotropic displacement parameters (Å^2^)

**Table:**

	*x*	*y*	*z*	*U*_iso_*/*U*_eq_	
Br	0.09369 (2)	0.119447 (19)	0.255278 (19)	0.03270 (8)	
S	0.83063 (5)	0.46517 (4)	0.59651 (5)	0.02459 (11)	
O1	0.81346 (14)	0.07754 (11)	0.46466 (12)	0.0215 (2)	
O2	1.24425 (15)	0.21464 (13)	0.82349 (12)	0.0254 (3)	
O3	1.00246 (16)	0.27962 (14)	0.82283 (14)	0.0330 (3)	
O4	0.73464 (19)	0.52767 (13)	0.49185 (15)	0.0335 (3)	
C1	0.7776 (2)	0.28864 (16)	0.52535 (17)	0.0202 (3)	
C2	0.6130 (2)	0.20245 (16)	0.44185 (16)	0.0195 (3)	
C3	0.4469 (2)	0.21889 (17)	0.39790 (17)	0.0222 (3)	
H3	0.4214	0.3025	0.4195	0.027*	
C4	0.3225 (2)	0.10378 (18)	0.32050 (17)	0.0230 (3)	
C5	0.3554 (2)	−0.02394 (17)	0.28541 (18)	0.0244 (3)	
H5	0.2667	−0.0978	0.2327	0.029*	
C6	0.5200 (2)	−0.04033 (17)	0.32914 (18)	0.0237 (3)	
H6	0.5453	−0.1240	0.3069	0.028*	
C7	0.6445 (2)	0.07386 (16)	0.40746 (17)	0.0202 (3)	
C8	0.8916 (2)	0.20979 (16)	0.53584 (17)	0.0203 (3)	
C9	1.0744 (2)	0.23516 (17)	0.61405 (18)	0.0225 (3)	
H9A	1.1371	0.3191	0.6023	0.027*	
H9B	1.1206	0.1619	0.5790	0.027*	
C10	1.0990 (2)	0.24522 (16)	0.76378 (18)	0.0226 (3)	
C11	1.2757 (2)	0.2174 (2)	0.96764 (19)	0.0344 (4)	
H11A	1.1845	0.1527	0.9803	0.041*	
H11B	1.2802	0.3073	1.0174	0.041*	
C12	1.4438 (2)	0.18093 (19)	1.01854 (19)	0.0301 (4)	
H12A	1.4417	0.0973	0.9584	0.036*	
H12B	1.4531	0.1625	1.1072	0.036*	
C13	1.6038 (2)	0.28719 (19)	1.03000 (19)	0.0290 (4)	
H13	1.5949	0.3064	0.9407	0.035*	
C14	1.6262 (3)	0.4197 (2)	1.1307 (2)	0.0483 (6)	
H14A	1.5316	0.4588	1.1004	0.072*	
H14B	1.7304	0.4819	1.1373	0.072*	
H14C	1.6307	0.4018	1.2180	0.072*	
C15	1.7591 (3)	0.2284 (3)	1.0724 (2)	0.0476 (6)	
H15A	1.8598	0.2922	1.0746	0.071*	
H15B	1.7440	0.1449	1.0085	0.071*	
H15C	1.7710	0.2111	1.1608	0.071*	
C16	0.7171 (3)	0.4627 (2)	0.7195 (2)	0.0363 (5)	
H16A	0.7307	0.5540	0.7680	0.055*	
H16B	0.7619	0.4109	0.7819	0.055*	
H16C	0.5982	0.4217	0.6743	0.055*	

### Atomic displacement parameters (Å^2^)

**Table:**

	*U*^11^	*U*^22^	*U*^33^	*U*^12^	*U*^13^	*U*^23^
Br	0.01875 (11)	0.04364 (13)	0.03281 (12)	0.00906 (8)	0.00246 (8)	0.00820 (8)
S	0.0252 (2)	0.01788 (19)	0.0290 (2)	0.00410 (15)	0.00857 (18)	0.00199 (16)
O1	0.0193 (6)	0.0199 (5)	0.0249 (6)	0.0061 (4)	0.0063 (5)	0.0035 (5)
O2	0.0212 (6)	0.0340 (6)	0.0203 (6)	0.0080 (5)	0.0042 (5)	0.0061 (5)
O3	0.0244 (7)	0.0439 (8)	0.0290 (7)	0.0089 (6)	0.0098 (6)	0.0008 (6)
O4	0.0438 (8)	0.0243 (6)	0.0391 (8)	0.0130 (6)	0.0164 (6)	0.0136 (6)
C1	0.0208 (8)	0.0177 (7)	0.0213 (8)	0.0042 (6)	0.0060 (6)	0.0038 (6)
C2	0.0215 (8)	0.0201 (7)	0.0174 (8)	0.0050 (6)	0.0060 (6)	0.0055 (6)
C3	0.0231 (8)	0.0240 (8)	0.0213 (8)	0.0084 (6)	0.0067 (7)	0.0076 (6)
C4	0.0171 (8)	0.0325 (9)	0.0201 (8)	0.0066 (6)	0.0048 (6)	0.0086 (7)
C5	0.0221 (8)	0.0270 (8)	0.0207 (8)	0.0005 (6)	0.0060 (7)	0.0034 (7)
C6	0.0265 (9)	0.0201 (8)	0.0243 (9)	0.0047 (6)	0.0100 (7)	0.0020 (6)
C7	0.0196 (8)	0.0227 (8)	0.0200 (8)	0.0068 (6)	0.0072 (6)	0.0061 (6)
C8	0.0208 (8)	0.0192 (7)	0.0206 (8)	0.0039 (6)	0.0070 (6)	0.0037 (6)
C9	0.0190 (8)	0.0235 (8)	0.0250 (9)	0.0063 (6)	0.0063 (7)	0.0053 (6)
C10	0.0178 (8)	0.0192 (7)	0.0264 (9)	−0.0002 (6)	0.0053 (7)	0.0015 (6)
C11	0.0268 (10)	0.0529 (12)	0.0216 (9)	0.0053 (8)	0.0065 (7)	0.0102 (8)
C12	0.0317 (10)	0.0338 (9)	0.0228 (9)	0.0054 (7)	0.0037 (7)	0.0109 (7)
C13	0.0263 (9)	0.0348 (10)	0.0224 (9)	0.0052 (7)	0.0021 (7)	0.0080 (7)
C14	0.0529 (14)	0.0353 (11)	0.0417 (13)	0.0034 (10)	0.0010 (11)	−0.0012 (10)
C15	0.0338 (12)	0.0684 (16)	0.0400 (13)	0.0200 (11)	0.0044 (10)	0.0130 (11)
C16	0.0512 (12)	0.0335 (10)	0.0317 (11)	0.0175 (9)	0.0215 (10)	0.0054 (8)

### Geometric parameters (Å, °)

**Table:**

Br—C4	1.9047 (17)	C9—C10	1.517 (2)
S—O4	1.4984 (14)	C9—H9A	0.9700
S—C1	1.7651 (16)	C9—H9B	0.9700
S—C16	1.796 (2)	C11—C12	1.509 (3)
O1—C7	1.374 (2)	C11—H11A	0.9700
O1—C8	1.3791 (19)	C11—H11B	0.9700
O2—C10	1.335 (2)	C12—C13	1.527 (3)
O2—C11	1.464 (2)	C12—H12A	0.9700
O3—C10	1.208 (2)	C12—H12B	0.9700
C1—C8	1.358 (2)	C13—C14	1.523 (3)
C1—C2	1.449 (2)	C13—C15	1.528 (3)
C2—C3	1.397 (2)	C13—H13	0.9800
C2—C7	1.403 (2)	C14—H14A	0.9600
C3—C4	1.384 (2)	C14—H14B	0.9600
C3—H3	0.9300	C14—H14C	0.9600
C4—C5	1.400 (2)	C15—H15A	0.9600
C5—C6	1.385 (2)	C15—H15B	0.9600
C5—H5	0.9300	C15—H15C	0.9600
C6—C7	1.381 (2)	C16—H16A	0.9600
C6—H6	0.9300	C16—H16B	0.9600
C8—C9	1.484 (2)	C16—H16C	0.9600
			
O4—S—C1	106.60 (8)	O2—C10—C9	111.26 (14)
O4—S—C16	105.52 (9)	O2—C11—C12	107.96 (15)
C1—S—C16	98.25 (9)	O2—C11—H11A	110.1
C7—O1—C8	106.47 (12)	C12—C11—H11A	110.1
C10—O2—C11	114.98 (14)	O2—C11—H11B	110.1
C8—C1—C2	107.39 (14)	C12—C11—H11B	110.1
C8—C1—S	124.39 (13)	H11A—C11—H11B	108.4
C2—C1—S	128.16 (12)	C11—C12—C13	116.26 (16)
C3—C2—C7	119.64 (15)	C11—C12—H12A	108.2
C3—C2—C1	135.88 (15)	C13—C12—H12A	108.2
C7—C2—C1	104.44 (14)	C11—C12—H12B	108.2
C4—C3—C2	116.29 (15)	C13—C12—H12B	108.2
C4—C3—H3	121.9	H12A—C12—H12B	107.4
C2—C3—H3	121.9	C14—C13—C12	112.39 (18)
C3—C4—C5	123.73 (15)	C14—C13—C15	109.96 (18)
C3—C4—Br	118.49 (13)	C12—C13—C15	108.72 (17)
C5—C4—Br	117.77 (13)	C14—C13—H13	108.6
C6—C5—C4	120.00 (15)	C12—C13—H13	108.6
C6—C5—H5	120.0	C15—C13—H13	108.6
C4—C5—H5	120.0	C13—C14—H14A	109.5
C7—C6—C5	116.59 (15)	C13—C14—H14B	109.5
C7—C6—H6	121.7	H14A—C14—H14B	109.5
C5—C6—H6	121.7	C13—C14—H14C	109.5
O1—C7—C6	125.46 (15)	H14A—C14—H14C	109.5
O1—C7—C2	110.78 (14)	H14B—C14—H14C	109.5
C6—C7—C2	123.75 (15)	C13—C15—H15A	109.5
C1—C8—O1	110.91 (14)	C13—C15—H15B	109.5
C1—C8—C9	133.32 (15)	H15A—C15—H15B	109.5
O1—C8—C9	115.60 (14)	C13—C15—H15C	109.5
C8—C9—C10	111.79 (14)	H15A—C15—H15C	109.5
C8—C9—H9A	109.3	H15B—C15—H15C	109.5
C10—C9—H9A	109.3	S—C16—H16A	109.5
C8—C9—H9B	109.3	S—C16—H16B	109.5
C10—C9—H9B	109.3	H16A—C16—H16B	109.5
H9A—C9—H9B	107.9	S—C16—H16C	109.5
O3—C10—O2	123.95 (17)	H16A—C16—H16C	109.5
O3—C10—C9	124.76 (16)	H16B—C16—H16C	109.5
			
O4—S—C1—C8	137.41 (15)	C1—C2—C7—O1	0.44 (18)
C16—S—C1—C8	−113.60 (16)	C3—C2—C7—C6	1.1 (2)
O4—S—C1—C2	−39.41 (17)	C1—C2—C7—C6	179.05 (16)
C16—S—C1—C2	69.57 (17)	C2—C1—C8—O1	0.16 (19)
C8—C1—C2—C3	177.04 (18)	S—C1—C8—O1	−177.22 (11)
S—C1—C2—C3	−5.7 (3)	C2—C1—C8—C9	−174.86 (17)
C8—C1—C2—C7	−0.36 (18)	S—C1—C8—C9	7.8 (3)
S—C1—C2—C7	176.90 (13)	C7—O1—C8—C1	0.11 (18)
C7—C2—C3—C4	−0.5 (2)	C7—O1—C8—C9	176.10 (14)
C1—C2—C3—C4	−177.59 (18)	C1—C8—C9—C10	73.0 (2)
C2—C3—C4—C5	−0.2 (2)	O1—C8—C9—C10	−101.84 (16)
C2—C3—C4—Br	−179.23 (12)	C11—O2—C10—O3	3.6 (2)
C3—C4—C5—C6	0.3 (3)	C11—O2—C10—C9	−178.09 (14)
Br—C4—C5—C6	179.34 (13)	C8—C9—C10—O3	−26.2 (2)
C4—C5—C6—C7	0.3 (2)	C8—C9—C10—O2	155.53 (14)
C8—O1—C7—C6	−178.93 (16)	C10—O2—C11—C12	−179.92 (14)
C8—O1—C7—C2	−0.35 (17)	O2—C11—C12—C13	70.7 (2)
C5—C6—C7—O1	177.40 (15)	C11—C12—C13—C14	62.3 (2)
C5—C6—C7—C2	−1.0 (2)	C11—C12—C13—C15	−175.75 (17)
C3—C2—C7—O1	−177.47 (14)		

### Hydrogen-bond geometry (Å, °)

**Table:**

*D*—H···*A*	*D*—H	H···*A*	*D*···*A*	*D*—H···*A*
C3—H3···O4^i^	0.93	2.56	3.437 (2)	157
C5—H5···O3^ii^	0.93	2.51	3.383 (2)	157
C9—H9A···O4^iii^	0.97	2.26	3.192 (2)	162
C9—H9B···O1^iv^	0.97	2.61	3.541 (2)	160

Symmetry codes: (i) −*x*+1, −*y*+1, −*z*+1; (ii) −*x*+1, −*y*, −*z*+1; (iii) −*x*+2, −*y*+1, −*z*+1; (iv) −*x*+2, −*y*, −*z*+1.
